# Embodied neurology: an integrative framework for neurological disorders

**DOI:** 10.1093/brain/aww076

**Published:** 2016-04-21

**Authors:** Patrick Freund, Karl Friston, Alan J. Thompson, Klaas E. Stephan, John Ashburner, Dominik R. Bach, Zoltan Nagy, Gunther Helms, Bogdan Draganski, Siawoosh Mohammadi, Martin E. Schwab, Armin Curt, Nikolaus Weiskopf

**Affiliations:** ^1^ Spinal Cord Injury Centre Balgrist, University Hospital Zurich, University of Zurich, Zurich, Switzerland; ^2^ Wellcome Trust Centre for Neuroimaging, UCL Institute of Neurology, University College London, London, UK; ^3^ Department of Brain Repair and Rehabilitation, UCL Institute of Neurology, University College London, London, UK; ^4^ Department of Neurophysics, Max Planck Institute for Human Cognitive and Brain Sciences, Leipzig, Germany; ^5^ Translational Neuromodeling Unit (TNU), Institute of Biomedical Engineering, University of Zurich and Swiss Federal Institute of Technology (ETH Zurich), Switzerland; ^6^ Max Planck Institute for Metabolism Research, Cologne, Germany; ^7^ Department of Psychiatry, Psychotherapy, and Psychosomatics, University of Zurich, Zürich, Switzerland; ^8^ Laboratory for Social and Neural Systems Research (SNS Lab), University of Zurich, Switzerland; ^9^ Department of Clinical Sciences, Lund University, Lund, Sweden; ^10^ Laboratoire de Recherche en Neuroimagerie (LREN), University of Lausanne, Department of Clinical Neurosciences, CHUV, Lausanne, Switzerland; ^11^ Department of Systems Neuroscience, University Medical Centre Hamburg-Eppendorf Hamburg, Germany; ^12^ Brain Research Institute, University of Zurich and Dept. of Health Sciences and Technology, ETH Zurich, 8057 Zurich, Switzerland

**Keywords:** biophysical models, multiscale interactions, neuroimaging, precision neurology, dynamic causal modelling

## Abstract

From a systems biology perspective, the brain and spinal cord are interwoven with the body: they are ‘embodied’. Freund *et al*. propose an integrative framework based on biophysical models that aims to characterize neurological disorders and minimize their impact on patients by considering functional interactions between supra-spinal, spinal and peripheral regions simultaneously.

## Graphical abstract

**Figure:**
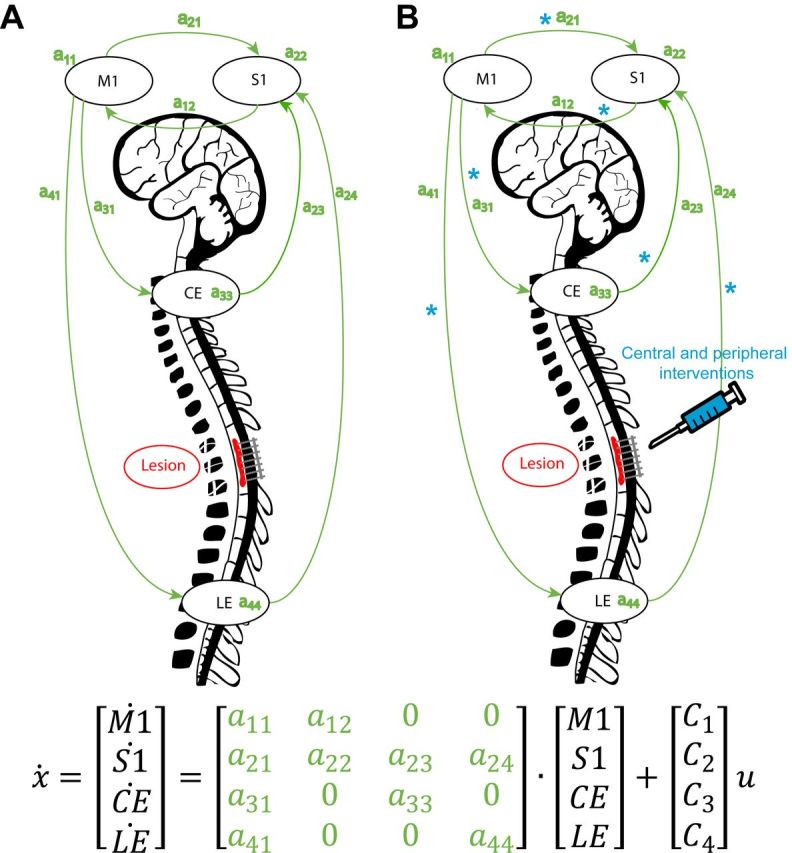
From a systems biology perspective, the brain and spinal cord are interwoven with the body: they are ‘embodied’. Freund *et al*. propose an integrative framework based on biophysical models that aims to characterize neurological disorders and minimize their impact on patients by considering functional interactions between supra-spinal, spinal and peripheral regions simultaneously.

## Introduction

From a systems biology perspective, the brain and spinal cord are interwoven with the body, through afferent and efferent synaptic connections—they are literally ‘embodied’ (Adams *et al.*, 2013). Neurologists appreciate the embodied nature of neurological disorders in terms of diagnosis, classification and their understanding of the underlying pathophysiology. They routinely use a combination of physical examinations (e.g. scales that test motor, sensory and autonomic function) in conjunction with physiological, biochemical and anatomical measures (e.g. electrophysiology, serum and CSF, and radiology) of the peripheral and central nervous system.

These measures often produce combinations of symptoms and signs that translate into conventional nosological classifications. While therapeutics focus on the ‘treatable’ cause of a disorder, it is difficult to separate out the impact on the patient due to the primary effects of a lesion/insult etc. and the effects of (possibly delayed) secondary processes that may be reasonable targets for interventions on their own. Moreover, standard neurological assessments often fail to distinguish between pathogenic and compensatory processes.

This state of affairs calls for a better understanding of neurological disease within a formal framework that links pathology to phenomenology (i.e. symptoms, impairment and physical signs). We suggest that such a framework should pay special attention to the embodied nature of the nervous system and the implicit pathophysiological and compensatory processes that can be present throughout the neuroaxis. In particular, we postulate that reciprocal information flows, between the body and the nervous system, are crucial for understanding and treating neurological disorders.

This framework aims to link pathology to phenomenology, while respecting the ‘embodied’ nature of the nervous system. If fully realized, the framework of embodied neurology has the potential to improve functional outcome following individualized treatment (i.e. precision neurology), promote successful translation of novel therapeutics into clinical use, and refine nosology in the context of disease heterogeneity.

Our description of embodied neurology is largely theoretical and is based on a series of focused workshops. It draws on recent advances in biophysical modelling of functional (Deco *et al.*, 2008) and microstructural processes and neuroimaging (Weiskopf *et al.*, 2015). These advances—together with preclinical research—constitute the three tenets of embodied neurology: biophysical modelling, quantitative physiological measures (with an emphasis on non-invasive neuroimaging) and preclinical research on basic mechanisms. These three have a particular focus on the entire nervous system.

## Embodiment and neurology

**Figure 1:**
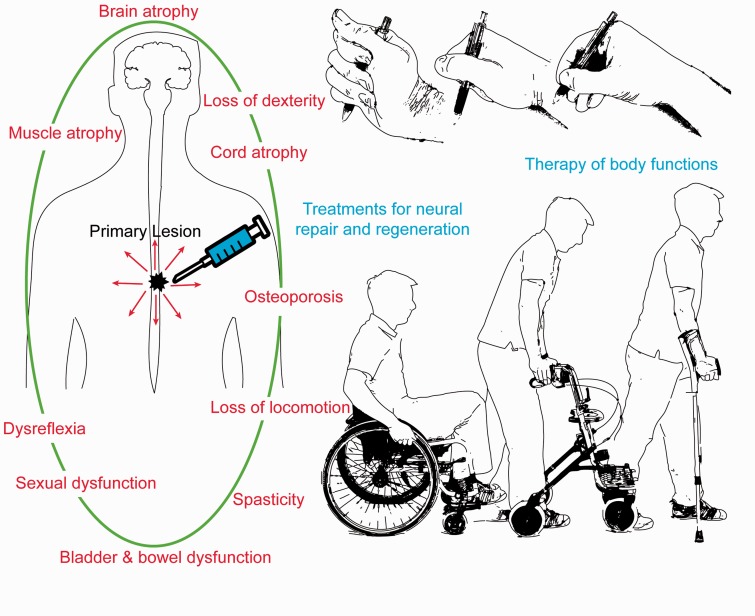
**The extensive sequelae following a focal spinal cord lesion.** Effects spanning the entire neuroaxis and periphery leading to spinal and cortical atrophy, paralysis, autonomic dysfunction and manifold functional impairments of the body (noticed as symptoms, signs and physical measures) are shown. To optimize functional recovery, treatments cannot be limited to restore the impaired two-way communication between the nervous system and the body but needs also to incorporate means to compensate for changes of the peripheral targets (joints, muscle fibre composition, osteoporosis, etc.).

The nervous system has a hierarchical (i.e. multi-level) structure of loops and recurrent processes that necessarily entails compensation, decompensation and the compounding of functional deficits (Jackson, 1958). Each level of the neuroaxis has distinct functions that contextualize lower levels: processing in lower levels (right down to primary afferents and efferents of the sensorimotor system) inform and enslave higher levels and vice versa (Adams *et al.*, 2013). In other words, the motor plant and peripheral nervous system induce neuronal responses and plasticity in the CNS, while the CNS modulates peripheral reflexes and coordinates movements. This is important because embodied symptoms themselves can confound neuropathology; by virtue of the circular causality implied by an embodied or enactivist view. For example, spinal cord injury and brain insult can lead to immediate impairment of sensorimotor control and autonomic dysfunction, which is followed by adaptations of skeletal muscle function and anatomy (i.e. changes in muscle tone, muscle fibre composition, fibre elastic properties, atrophy etc.), bone density (eventually osteoporosis) and changes in cardiovascular performance or control of internal organs (Fig. 1). Clinical observations typically reveal that downstream plasticity associated with functional improvements below complete or subcomplete spinal cord lesions remains ineffective (Huber *et al.*, 2015). Clinically less appreciated are consequences of lesion-induced plasticity in the reverse (upstream) direction—i.e. deafferentation of cortical regions (due to loss or impaired sensory and proprioceptive inputs)—although they may show marked neuropathological consequences, including cortical atrophy, plastic cortical map changes, or axonal retraction (Huber *et al.*, 2015).

The embodied nature of the complex interactive sensorimotor system suggests that even successful repair of focally damaged fibre tracts through a regenerative treatment [like anti-Nogo-A antibodies in spinal cord injury (Huber *et al.*, 2015)] may not lead to complete recovery. This is because even though tissues and organs are exterior to the CNS they are inevitably impacted upon by the consequences of the central lesion (e.g. muscle atrophy, osteoporosis, bladder and bowel dysfunction, etc.). Crucially, these require additional interventions (e.g. physio/occupational-therapy, bladder-bowel management, etc.) to overcome consecutive symptoms and impairment and to adjust the functional networks formed by the newly growing fibres and integrate them in a functionally meaningful way with the periphery as well as the higher CNS centres (Fig. 1 provides a schematic example).

Thus two-pronged treatments—targeting the central and peripheral nervous system and the bodily functions they serve—may be necessary to re-establish a functional interaction between the body and brain.

This overarching approach has already found traction in computational approaches to the interactions between supra-spinal and spinal regions in pain and placebo hypoalgesia (Büchel *et al.*, 2014) and has been considered for soft neurological signs in schizophrenia, e.g. abnormal slow pursuit eye movements (Adams *et al.*, 2012). The latter example is particularly interesting because a formal (i.e. computational) understanding of oculomotor control enables one to model both the central (neurocomputational) and embodied (eye movement) aspects of oculomotor control and quantify the effect of one on the other. In principle, this enables assays of synaptic neuromodulation based purely on peripheral measures (e.g. slow pursuit eye movements in schizophrenia).

In the same vein, there is an inherent circular causality in many neurological conditions. For example in epilepsy (Cooray *et al.*, 2016), seizure activity can be triggered by peripheral stimuli, such as stress, sleep deprivation, photic flicker, etc. In turn, seizure activity may preclude behaviours that protect from epileptogenic triggers. A more subtle example of circular causality in epileptogenesis follows from the embodiment of fast neuronal activity in a metabolic (i.e. systemic) or neuronal milieu. For example, current computational models of seizure activity emphasize the circular causality between fast (neuronal) and slow timescales, where slow fluctuations (e.g. in extracellular potassium) affect the neuron membrane potential and are affected by neuronal activity through processes such as activity-dependent plasticity. The implicit separation of timescales has motivated recent advances in dynamic causal modelling (see below) to track slow fluctuations that predispose to fast seizure activity (Cooray *et al.*, 2016).

Similarly, a neurophysiologic-metabolic imbalance has been associated with the phenomenon of burst suppression. Burst suppression is an electroencephalogram (EEG) pattern reflecting synchronized thalamic discharges that can occur in hypoxic and anaesthetic encephalopathy (e.g. Ching *et al.*, 2012). Burst suppression is a dynamic process modulated by the lowering of extracellular calcium concentrations to levels that inhibit neuronal activity. This results in suppression periods, during which the calcium ion concentrations are restored to normal levels by neuronal pumps, thus causing the cortex to resume bursting behaviour. In short, an inability to properly regulate extracellular calcium levels (due to a pathologically altered blood–brain permeability) produces shorter burst periods and augments suppression periods. Dynamical processes of this sort are amenable to quantitative modelling. In principle, this means that they can be used as a basis for quantitative assays of pathophysiology, provided we have sufficiently detailed and valid biophysical models.

## Modelling of disease processes

Embodied neurology attempts to account for the circular causality inherent in coupled dynamical systems by measuring and modelling biophysical functional interactions across multiple levels within the interlinked central and peripheral nervous system—as well as the bodily functions they serve. This theme is especially prescient for neurology because it speaks to distributed changes at several spatial and temporal scales. For example, a focal traumatic lesion in the spinal cord can have far-reaching consequences both in terms of cortical reorganization at distant sites, such as functional diaschisis and functional as well as architectural changes within the spinal cord itself (Huber *et al.*, 2015). There are numerous examples of biophysical modelling in neuropsychiatry, drawing on dynamic causal or other (neural mass or mean field) models (e.g. Deco *et al.*, 2008). An interesting example here is the use of biophysical models of distributed processing to explain functional MRI data in terms of remote diaschisis effects, not on neuronal responses, but on neuronal connectivity *per se*. Embodied neurology hopes to generalize this modelling approach to include peripheral measures as an explicit part of biophysical models.

## Implications of embodied neurology for treatment of neurological disorders

Exogenous modulation of network dynamics can improve performance and symptomatology. For example, a single anodal transcranial direct current stimulation to the left inferior frontal gyrus (Meinzer *et al.*, 2013) improved performance during overt semantic word generation—a task that is affected adversely by age. Crucially, this improvement was directly linked to changes in neuronal network connectivity as assessed by multimodal neuroimaging in the elderly.

In addition to focusing on primary processes at the site of the initial lesion, targeting areas affected by secondary processes (like diaschisis) could potentially offer a wider therapeutic window, as there is often a time lag between the primary insult (e.g. trauma, inflammation, etc.) and secondary processes (e.g. network reorganization) that may involve a range of cellular processes. Treatments targeting secondary processes in one neurological disorder may be repurposed for another, offering novel treatment options.

## Challenges and future developments

To achieve the objectives of embodied neurology, several challenges in functional and structural biophysical modelling, neuroimaging and clinical measures need to be met. We will consider these challenges in terms of the three tenets of embodied neurology:

### Computational models of functional processes

The central tenet of this framework relies on formulating mechanistic hypotheses about how peripheral processes (visible as signs, symptoms and laboratory measures to the clinician’s eye) translate into an embodied central response. Biophysical models of interacting central and peripheral systems can provide a mechanistic and quantitative characterization of physiology as well as pathology. Model-based indices (e.g. time and frequency domain responses and dynamic structural changes) not only furnish mechanistic insights into a distributed pathology but might also serve as more powerful clinical predictors than (level specific) local measurements (for a demonstration of this in stroke research, see Brodersen *et al.*, 2011).

**Figure 2:**
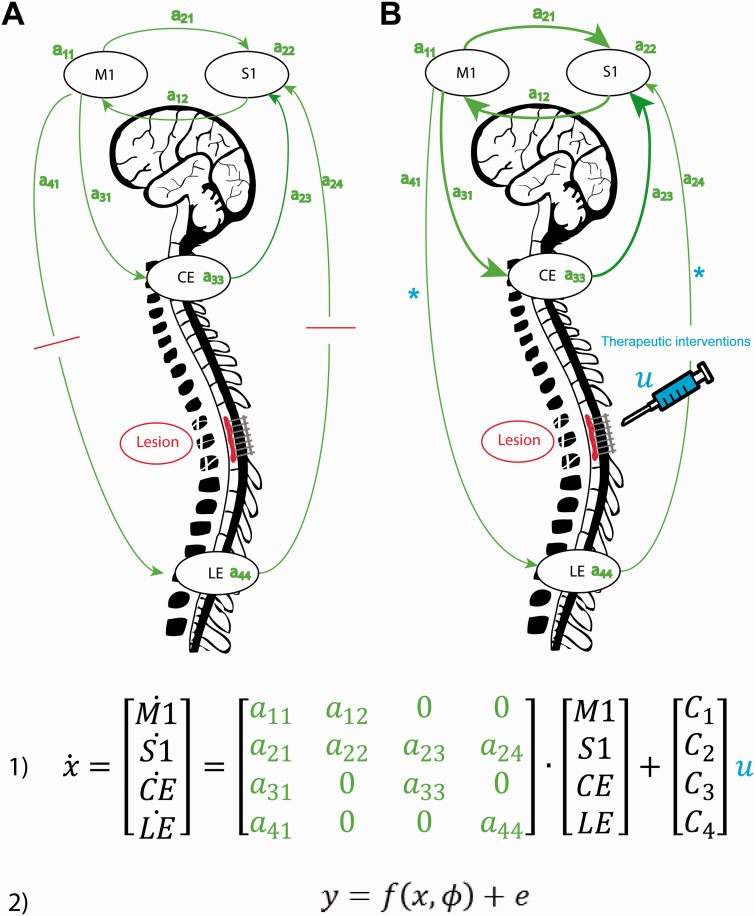
**Integrating multiscale interactions into biophysical models** (**A**) Dynamic causal modelling of neuroimaging data can be used for neuronal system identification to assess distributed changes at several spatial scales across the nervous system. This example presents a very simple model of how the distant effects of a focal spinal lesion on interactions among cortical areas can be modelled. The figure depicts a minimal system involving the primary motor (M1) and primary sensory (S1) cortices as well as cervical (CE) and lumbar (LE) enlargements of the spinal cord. In dynamic causal modelling, this system is described as a weighted, directed graph, where nodes represent regional neuronal population activity (which cannot be observed directly). These ‘hidden neuronal states’ influence each other through directed synaptic connections (effective connectivity, quantified by coupling parameters *a*_11_…*a*_44_) and may be additionally influenced by external experimental manipulations *u*, such as sensory stimuli, motor commands, or central and peripheral treatments (**B**), with parameters *u*. These mechanisms can be described as a set of ordinary differential equations (Equation 1). Note the directionality of the graph: M1 directly affects spinal cord components due to descending neuronal tracts, while influences in the reverse direction are relayed via S1. The experimental measurement *y* obtained below and above the lesion is predicted by an observation equation (Equation 2) that maps the hidden neuronal states to observations (blood oxygen level-dependant functional MRI in this case). A model like the one shown above enables one to obtain quantitative indices of cortical plasticity (changes in effective connectivity) in response to a distal spinal cord lesion. These indices not only reveal mechanisms underlying supra-spinal pathology, but might also serve useful as predictors for clinical variables (Brodersen *et al.* 2011). (**B**) For example a complete lesion at the thoracic level results in the complete loss of effective connectivity of efferent and afferent information flow (*a*_41 _= 0, *a*_24 _= 0), but a restorative treatment aiming at the repair of the spinal cord is expected to increase the coupling between these regions (blue asterisk) and beyond (thick lines).

One potentially fruitful approach is the (dynamic) causal modelling of the coupling between the central and peripheral systems (Adams *et al.*, 2013). Dynamic causal modelling (DCM) combines a model of neuronal population dynamics with a forward model to measureable neuronal signals like functional MRI, EEG and magnetoencephalography (MEG). This enables one to infer functional coupling (or more precisely effective connectivity) among different components of the central and peripheral nervous systems from non-invasive data such as functional MRI, EEG, MEG or structural data. The idea here is to assess to what extent coupling between these regions is influenced by pathology (e.g. level of injury, time since injury or experimental intervention). As illustrated by the example in Fig. 2, this type of biologically grounded modelling can be used to quantify cortical plasticity (indexed by changes in effective connectivity) in response to a spinal cord lesion but also to peripheral quantifiable symptoms.

For electrophysiological data, different DCMs exist that take the form of neural mass or mean field models (for a taxonomy of models, see Deco *et al.*, 2008). Depending on the questions to be addressed, more detailed models, such as neural field models may be used, providing additional insights into disconnections and their synaptic mechanisms. Indeed, DCM has already been established as a non-invasive assay of synaptic function, which may be particularly useful in a neurogenetic setting. The sorts of biophysical models used in DCM have been shown to reliably describe many biophysical processes that are implicated in pathophysiology (e.g. short-term plasticity of glutamatergic synapses, spike-frequency adaptation). The choice of the appropriate biophysical model is usually based on the hypothesized neuronal processes of interest, and the temporal and spatial scales that are usefully informed by data.

### Advances in neuroimaging can unify structural and functional biopyhsical models

Recent advances in neuroimaging have transformed conventional imaging into a quantitative measurement tool for non-invasive measurements at synaptic timescales (with functional MRI) and even *in vivo* histology based on structural MRI (Weiskopf *et al.*, 2015). *In vivo* histology is based on ultra-high resolution multi-contrast MRI. This advance promises a detailed assessment of neuronal plasticity, including myelination, re-/demyelination, as well as changes to the axonal *g*-ratio. MRI served as a proxy for myelination and *g*-ratio, axon diameter and grey matter microstructure. In short, functional and structural MRI parameters can now provide important constraints for the functional biophysical modelling of neuronal dynamics. An important example here is non-invasive measures of myelination and its effects on spike time-dependent plasticity and conduction velocities. Provisional studies have started to combine dynamic causal models of axonal delays with *in vivo* histology measures of myelination. This is another example of the embodied brain that has clear relevance for demyelination diseases, and beyond.

Crucially, subject-specific structural data of this sort can be used to define anatomically informed priors, which constrain and individualize models of neuronal dynamics. For example, dynamic causal models of functional MRI were successfully informed and enhanced using diffusion-weighted imaging data describing anatomical connectivity (Stephan *et al.*, 2009). Finessing biophysical models by anatomical information becomes particularly important in the context of embodied neurology, which has to contend with long axonal conduction delays in sensorimotor control (Bojak and Liley, 2010).

### From bench to bedside

Preclinical studies are required to inform, constrain and validate the computational modelling in any neurological disorder. Such studies have demonstrated that features of neurodegeneration, including myelin, axonal and synaptic loss as well as functional impairments can be modulated by treatments of peripheral targets (e.g. tissues and organs outside the CNS), thereby offering new approaches to therapeutic intervention and highlighting the embodied character of the nervous system. Moreover, progress continues in the development of reparative and neuroprotective interventions to enhance recovery in many diseases.

Preclinical models have proven essential for elucidating disease mechanisms and for evaluating the extent of damage and the effects of novel treatment interventions. Recent methodological advances have enabled the invasive and non-invasive detection of functional and microstructural changes at single neuron and synapse levels with a temporal resolution in the millisecond range. Many of these parameters have been identified to link structural and functional changes to outcome in motor and sensory impairment. To improve the translation of knowledge from animal models to humans, the establishment of quantifiable and specific biomarkers in animal models and humans will be essential to establish the validity of these models in the setting of disease stage and treatment interventions.

## Conclusion

Embodied neurology promises insights into the multiscale interactions across the entire neuroaxis through unified biophysical models of structure and function as well as advanced neuroimaging techniques. It may provide the basis for a better understanding of the changes in neural control and plasticity that occur across the embodied central and peripheral nervous systems under physiological and pathological conditions. Thus, embodied neurology may be well placed to finesse nosological classification, to optimize therapeutic outcome by providing specifically targeted interventions and improve clinical diagnosis in the context of heterogeneity. If fully realized, embodied neurology will enable (i) the detection of beneficial plasticity versus detrimental changes; (ii) the utilization of high-resolution imaging of the entire nervous system; (iii) more specific characterization of structural and functional changes as they relate to tissue and connectivity changes; and (iv) the identification (and simulation) of optimal treatment processes for rehabilitation.

## References

[aww076-B13] Adams RA Perrinet LU Friston K. Smooth pursuit and visual occlusion: active inference and oculomotor control in schizophrenia. PLoS One 2012; 7: e47502. 23110076 10.1371/journal.pone.0047502 PMC3482214

[aww076-B1] Adams RA Shipp S Friston KJ. Predictions not commands: active inference in the motor system. Brain Struct Funct 2013; 218: 611–43. 23129312 10.1007/s00429-012-0475-5 PMC3637647

[aww076-B2] Bojak I Liley DTJ. Axonal velocity distributions in neural field equations. PLoS Comput Biol 2010; 6: e1000653. 20126532 10.1371/journal.pcbi.1000653 PMC2813262

[aww076-B3] Brodersen KH Schofield TM Leff AP Ong CS Lomakina EI Buhmann JM, Generative embedding for model-based classification of fMRI data. PLoS Comput Biol 2011; 7: e1002079. 21731479 10.1371/journal.pcbi.1002079 PMC3121683

[aww076-B4] Büchel C Geuter S Sprenger C Eippert F. Placebo analgesia: a predictive coding perspective. Neuron 2014; 81: 1223–39. 24656247 10.1016/j.neuron.2014.02.042

[aww076-B5] Ching S Purdon PL Vijayan S Kopell NJ Brown EN. A neurophysiological-metabolic model for burst suppression. Proc Natl Acad Sci USA 2012; 109: 3095–100. 22323592 10.1073/pnas.1121461109 PMC3286963

[aww076-B6] Cooray GK Sengupta B Douglas P Friston K. Dynamic causal modelling of electrographic seizure activity using bayesian belief updating. Neuroimage 2016; 125: 1142–54. 26220742 10.1016/j.neuroimage.2015.07.063 PMC4692455

[aww076-B7] Deco G Jirsa VK Robinson PA Breakspear M Friston K. The dynamic brain: from spiking neurons to neural masses and cortical fields. PLoS Comput Biol 2008; 4: e1000092. 18769680 10.1371/journal.pcbi.1000092 PMC2519166

[aww076-B8] Huber E Curt A Freund P. Tracking trauma-induced structural and functional changes above the level of spinal cord injury. Curr Opin Neurol 2015; 28: 365–72. 26110798 10.1097/WCO.0000000000000224

[aww076-B9] Jackson JH. Selected writings of Hughlings Jackson: Vol. 1. On epilepsy and epileptiform convulsions. Vol. 2. Evolution and dissolution of the nervous system; speech; various papers, addresses and lectures. Oxford, Basic Books; 1958 p. 510.

[aww076-B10] Meinzer M Lindenberg R Antonenko D Flaisch T Flöel A. Anodal transcranial direct current stimulation temporarily reverses age-associated cognitive decline and functional brain activity changes. J Neurosci 2013; 33: 12470–8. 23884951 10.1523/JNEUROSCI.5743-12.2013 PMC6618670

[aww076-B11] Stephan KE Tittgemeyer M Knösche TR Moran RJ Friston KJ. Tractography-based priors for dynamic causal models. Neuroimage 2009; 47: 1628–38. 19523523 10.1016/j.neuroimage.2009.05.096 PMC2728433

[aww076-B12] Weiskopf N Mohammadi S Lutti A Callaghan MF. Advances in MRI-based computational neuroanatomy: from morphometry to *in vivo* histology. Curr Opin Neurol 2015; 28: 313–22. 26132532 10.1097/WCO.0000000000000222

